# A Giant Parathyroid Adenoma Presenting With Parathyroid Crisis

**DOI:** 10.7759/cureus.43129

**Published:** 2023-08-08

**Authors:** Sara Esteves-Ferreira, Leonor Rodrigues, Rosa Dantas, Márcia Alves, Joana Guimarães

**Affiliations:** 1 Endocrinology Department, Centro Hospitalar do Baixo Vouga, Aveiro, PRT

**Keywords:** primary hyperparathyroidism, giant parathyroid adenoma, acute parathyroidism, parathyroid storm, parathyroid crisis

## Abstract

Giant parathyroid adenomas (GPA) are a benign cause of primary hyperparathyroidism (PHPT) that might present similarly to parathyroid carcinomas (PC). Rarely, PHPT can present with a parathyroid crisis, a life-threatening decompensation with severe hypercalcemia.

A 77-year-old woman presented with lethargy and muscle weakness. Investigation revealed parathyroid hormone-dependent hypercalcemia and an enlarged parathyroid measuring 31x24 mm. The patient was submitted for parathyroidectomy. Histology showed no evidence of malignancy, confirming a GPA.

We report a GPA presenting with a parathyroid crisis. The clinical picture mimicked that of a PC. There are no clinical, analytical, or imagiological features pathognomonic of PC.

## Introduction

Primary hyperparathyroidism (PHPT) is one of the most common endocrine disorders, mostly caused by benign parathyroid adenomas (PA) but can arise from parathyroid carcinoma (PC) in 1-2% of cases ​[1]. Giant parathyroid adenomas (GPA) represent a rare form of PA, with significant overlap in presentation with PC but with a benign course​ [2]. Differential diagnosis is paramount to ensure adequate treatment since PC should be resected en bloc, differently than PA, to ensure optimal prognosis ​[1]. However, a distinction between these conditions is not possible based solely on clinical, biochemical, or imagiologic findings, seeing that diagnosis of PC can only be confirmed through histopathology ​[1]. Rarely, PHPT can present with a parathyroid crisis, a life-threatening, acute hypercalcemic decompensation ​[3]. 

## Case presentation

A 77-year-old woman was admitted to the emergency room for muscle weakness and fatigue, associated with generalized muscle pain and constipation. The patient had a history of arterial hypertension, peptic ulcer disease, and multiple fractures. No past history of osteoporosis or kidney stones was described. 

A thorough review of the patient history revealed that a hypercalcemic hyperparathyroidism (calcium 12.76 mg/dL (reference range (RR) 8.7-10.4), PTH >2000 pg/mL (RR 19-88), creatinine 1.25 mg/dL (RR 0.5-1.1)) had been identified 8 months prior, during an admission due to a femur fracture requiring placement of a left hip prosthesis, but no follow-up was granted at the time. 

Physical examination revealed dehydration, lethargy, generally diminished muscle strength (4/5 grade in all limbs), and hyporeflexia. Vital signs were stable. No visible or palpable masses were detected on cervical examination, nor was hoarseness. Serum laboratory tests revealed: total calcium 13.44 mg/dL, albumin 4.0 g/dL (RR 3.4-4.8), ionized calcium (direct measurement) 1.92 mmol/L (RR 1.13-1.32), creatinine 3.37 mg/dL, parathyroid hormone (PTH) 2462 pg/mL, phosphorus 4.9 mg/dL (RR 2.4-5.1), magnesium 1.91 mg/dL (RR 1.3-2.7), vitamin D 8.4 ng/mL (RR 30-100), alkaline phosphatase (ALP) 908 U/L (RR 46-116), hemoglobin 6.9 g/dL (RR 11.5-16.5). Urinalysis revealed leukocyturia. Diagnoses of hypercalcemic hyperparathyroidism and urinary tract infection were made, the patient was admitted to the medical ward and started on antibiotics, IV fluids, and loop diuretics. 

**Figure 1:**
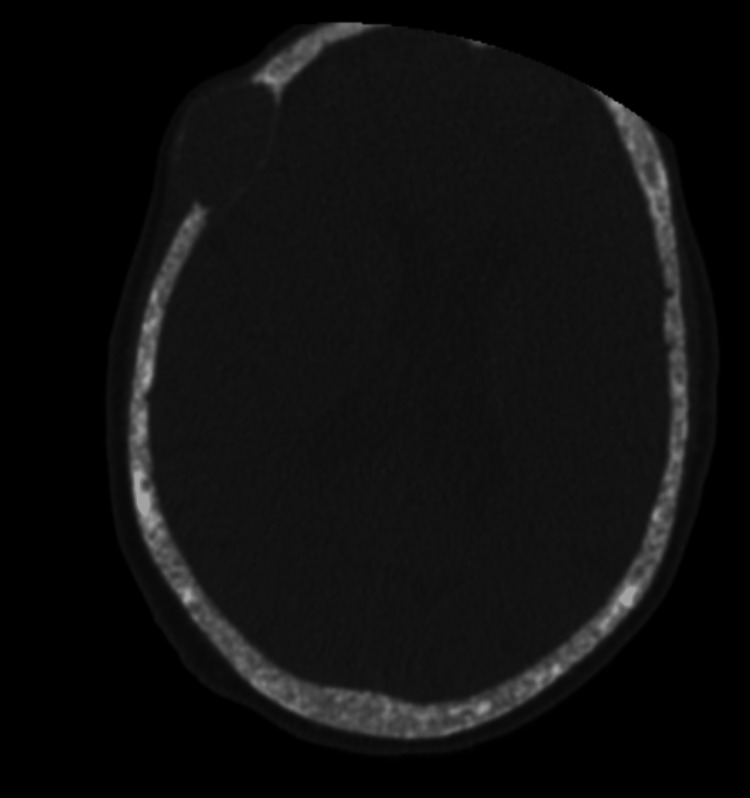
Salt and pepper skull on CT.

Abdominal ultrasound did not reveal kidney stones and cervical ultrasound didn’t locate any image compatible with enlarged parathyroid glands. Computed tomography (CT) showed multiple lytic bone lesions on the skull (Figure 1), costal arches, vertebral bodies, pubic branch, and humerus, consistent with brown tumors, a “salt and pepper” appearance of the skull and “rugger jersey spine” sign, as well as humeral chondrocalcinosis.

**Figure 2:**
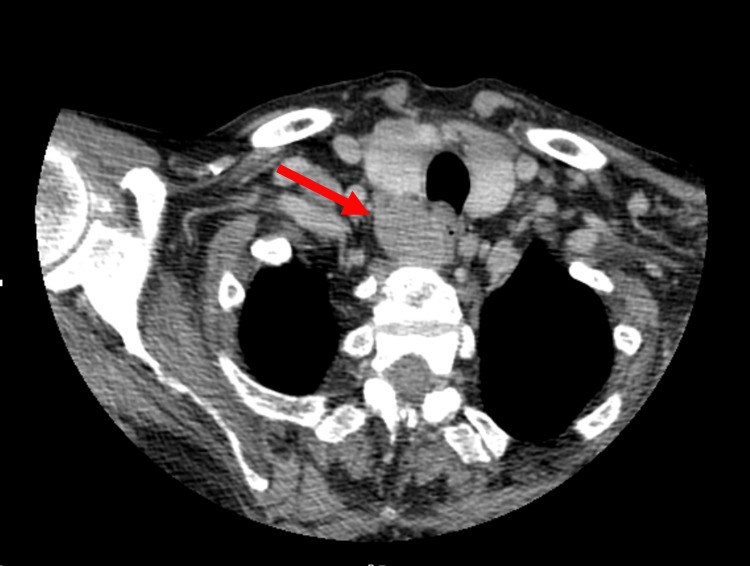
CT image showing the cervical lesion posterior (arrow) to the right thyroid lobe, measuring 31x24 mm and without apparent cleavage plane with the thyroid or esophagus.

**Figure 3:**
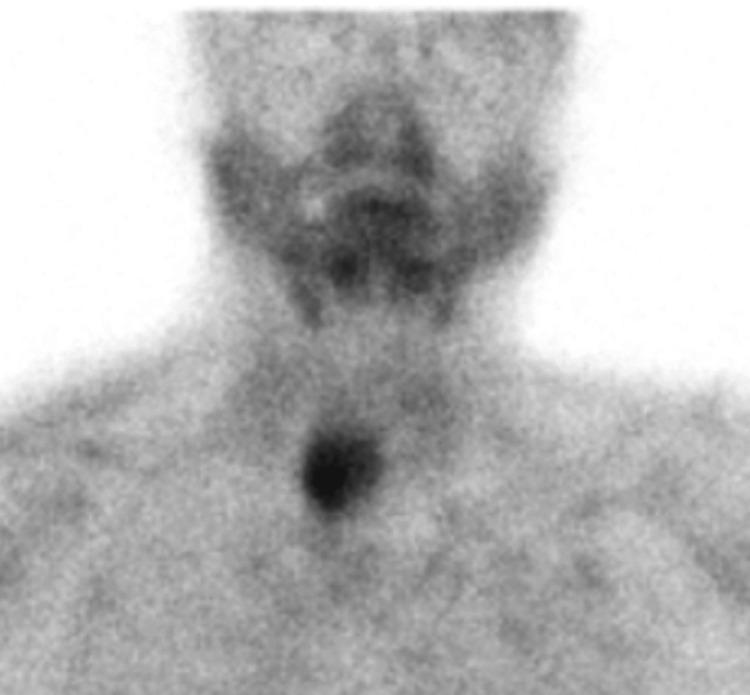
Sestamibi parathyroid scintigraphy showing an area of increased uptake adjacent to the right inferior thyroid pole.

A cervical expansive lesion was described posterior to the right thyroid lobe, with no apparent cleavage plane with the thyroid or esophagus (Figure 2), with 31x24 mm and spontaneous density of 40 HU, and multiple enlarged cervical lymph nodes at levels IIa, IIb, and III. Sestamibi parathyroid scintigraphy showed an extensive area of increased uptake adjacent to the right inferior thyroid pole (Figure 3). 

**Figure 4:**
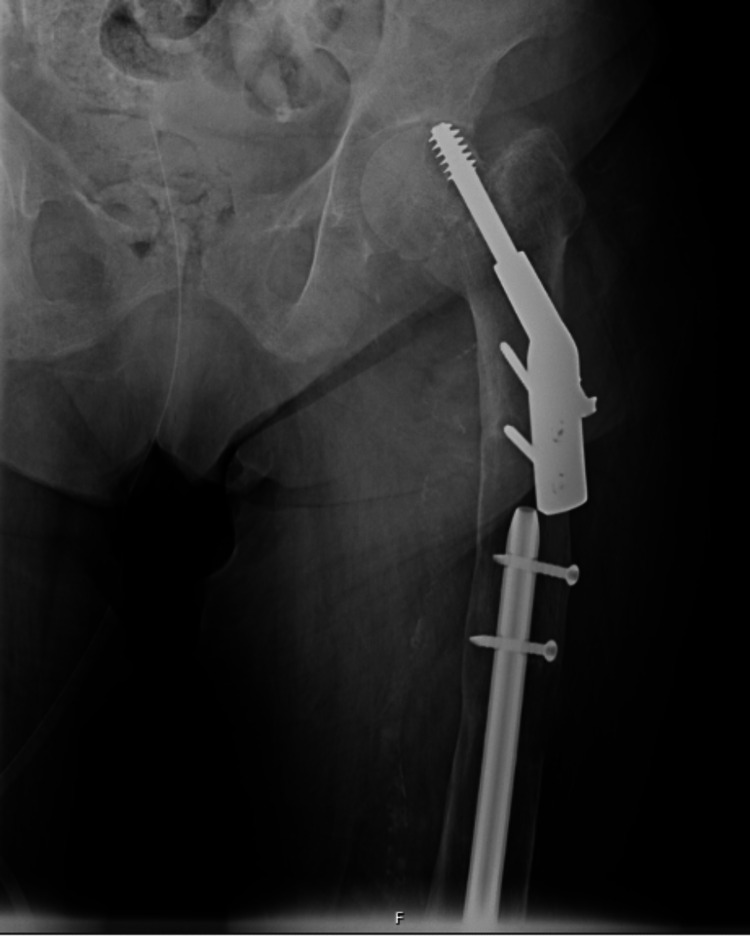
Radiograph of the left hip showing prosthetic dislocation.

Treatment with the calcimimetic cinacalcet was initiated at the dose of 30 mg twice daily since immediate surgery was not feasible. Vitamin D correction with oral cholecalciferol (0.1 mg daily) ensued. As the symptoms improved, pain and functional impotence of the left hip persisted, hip radiography showed displacement of the hip prosthesis (Figure 4) and elective hip surgery was scheduled. Neurologic status was restored, with the patient being alert, oriented, and with grade 5/5 muscle strength; kidney function improved (creatinine 1.43 mg/dL); and the patient was discharged while awaiting parathyroidectomy. Shortly after, the patient was again admitted due to a traumatic diaphyseal femur fracture and subjected to orthopedic surgery. 

The patient was subsequently submitted to parathyroidectomy. No suggestive features of malignancy were identified intraoperatively, including on frozen section analysis, so a localized excision was performed. Intraoperative PTH decreased from 1774 pg/mL at baseline to 446 and 296 pg/mL at 5 and 10 minutes, respectively. The histological study showed a parathyroid gland weighing 12 g and measuring 4.7x4x2.5 cm, with no suggestive features of carcinoma. After surgery, treatment with cinacalcet was discontinued and calcium and PTH levels improved (8.8 mg/dL and 105 pg/mL, respectively). 

## Discussion

Parathyroid crisis, also designated as acute parathyroidism or parathyroid storm, represents a severe presentation of hypercalcemic PHPT, with metabolic encephalopathy as the predominant feature, associated with significant dehydration and gastrointestinal symptoms. Renal and cardiovascular dysfunction may also be present ​[4,5]​. It frequently occurs after a period of mild, asymptomatic hypercalcemia, triggered by concurrent illness, dehydration, immobilization, or drugs such as thiazide diuretics ​[3]. This presentation can occur in up to 7% of patients with severe sporadic PHPT, more often in patients with PC ​[5]. Reports of mortality rates vary from 100% in earlier series to 7% in more recent works, and timely diagnosis and treatment are essential for successful outcomes ​[3]. 

Laboratory evaluation is remarkably characterized by very high PTH and calcium levels, resembling PC in biochemical terms, but more importantly than calcium levels, the acute nature of the symptoms and clinical instability are the cardinal features for defining the emergent nature of this clinical entity ​[4]. Indeed, even though hypercalcemic crises usually occur with very high calcium levels (>14 mg/dL), no cutoff value for this diagnosis was established ​[3,4]. Additionally, one should consider that total calcium alone might not be sufficient for evaluating calcium status. Discordant total and ionized calcium levels can be found in situations like critical illness or renal failure and especially in situations of calcium imbalance ​[6]. One could infer that measurement of ionized calcium could be beneficial whenever clinical manifestations of hypercalcemia are not congruent with total calcium levels. 

In this case, despite the modest elevation in total calcium levels, hypercalcemia presented acutely with multiorgan failure - lethargy, muscle weakness, dehydration, constipation, and acute kidney injury. Once ionized calcium was measured, it confirmed a more severe hypercalcemia than previously thought. This presentation can be framed within a parathyroid crisis, probably in the setting of urinary tract infection and immobilization from prosthesis dislocation. 

Early diagnosis can impact the prognosis of PHPT. The increased risk of death seen in patients with PHPT relates more strongly to glandular weight than to preoperative serum calcium levels. This finding highlights the importance of timely treatment before significant growth occurs ​[7]. 

A GPA is defined as a PA that is >3.5 g. Compared to other benign causes of PHPT, patients with GPA present with higher serum calcium and PTH levels, which correlate to gland size. Hence, GPA presentation resembles that of PC. However, GPAs don't appear to be associated with higher rates of persistent or recurrent PHPT, meaning prognosis is similar to other PAs, rather than that of PC ​[2].

Identification of patients suspected of PC is paramount, given that optimal outcomes require complete tumor resection at the time of initial surgery ​[8]. However, differential diagnosis with PA is challenging. 

In PHPT, clinical features result from hypercalcemia. PC is no exception, seeing that symptoms of organ invasion are uncommon ​[8]. Unlike in this case, GPAs are more often asymptomatic, possibly due to a more insidious onset of hypercalcemia, while PCs frequently present with symptomatic hypercalcemia ​[2,9]. Renal involvement in PC occurs in 32-60% of patients, and renal insufficiency was reported in up to 84%, contrasting with benign PHPT, where the prevalence of renal involvement is less than 20% ​[8]. Overt radiological signs of bone disease, such as subperiosteal bone resorption, a “salt and pepper” appearance of the skull, bone cysts, and brown tumors of the bones, are also more common in PC (44-91%) than in PA (<5%) ​[8]. Simultaneous renal and bone involvement is suggestive of PC, as well as pancreatitis, anemia, and peptic disease ​[8,9]. Additional clinical cues, such as a palpable cervical mass or vocal cord paralysis should also raise suspicion for PC ​[8,10]. 

Laboratory findings can provide additional clues as to the malignant nature of the disease. Serum calcium appears to be significantly higher in patients with PC than in those with PA. Some series have reported calcemia >14 mg/dL in 65-75% of patients with PC, but rates as low as 28% have also been described ​[9,10]. Robert et al. reported average calcium levels of 13.83 ± 4.21 mg/dL in patients with PC versus 11.58 ± 1.32 mg/dL in patients with PHPT of benign origin. Nevertheless, and not surprisingly given the significant overlap between the two entities, reported sensitivity and positive predictive value of serum calcium levels (56% and 14%, respectively) are insufficient to allow for an accurate identification of patients with PC ​[9]. Differences in PTH levels between these groups seem to be more dramatic: in PC, PTH levels average 10 times the upper level of normal, in contrast with 2.6 times in benign PHPT. Increases in PTH levels below four times the upper limit of normal are unlikely to correspond to PC ​[9]. ALP levels are also higher in patients in PC than in PA ​[8]. According to Bae et al., an ALP greater than 285 U/L predicted PC with 83% sensitivity and 97% specificity ​[11]. 

Cervical ultrasound has been shown to have low specificity for differentiating PC from PA, given the much higher prevalence of benign compared to malignant disease [12]. Some parathyroid features were proposed as suggestive of malignancy, including irregularity, heterogeneity, a depth/with ratio ≥1, lack of deformability, radially vascularization with no clear supplying vessels, and the appearance of a thickened capsule ​[12,13]. Infiltration of surrounding tissue and calcifications have a 100% positive predictive value for PC ​[13]. Size can also provide a clue as to the malignancy risk - a tumor size greater than 3 cm could predict PC with 91% sensitivity and 92% specificity, as reported by Bae et al. [11].​ Fine-needle aspiration is not recommended, given the risk of rupture of the tumor capsule and seeding on the biopsy track ​[8]. 

The treatment of benign and malignant diseases is also different. Although en bloc resection is the gold standard for the treatment of PC, over 50% of surgeries for PC remain simple local excision, a technique associated with significantly increased recurrence and mortality rates relating to the risk of capsule rupture, tumor spillage, and local seeding ​[1,8,10,14]. Conversely, GPAs appear to be able to be safely excised through minimally invasive surgery, with potentially lower perioperative complication rates ​[15-17]. However, robust data are lacking regarding the impact of more extensive surgery on complications, including hypoparathyroidism and recurrent laryngeal nerve palsy ​[18]. 

Diagnosis confirmation of PC requires histopathological analysis. According to the World Health Organization, infiltrative growth or histological proof of vascular invasion represent the minimum criteria for diagnosing PC ​[1]. As described by Schantz and Castleman, the histologic features indicative of PC are: a trabecular pattern, mitotic figures, thick fibrous bands, and capsular or vascular invasion ​[19]. Unfortunately, several of these features can be seen in PA ​[20]. An intraoperative frozen section examination could be helpful but, in the absence of clear evidence of invasive disease, it also frequently results in misdiagnosis ​[8,9]. This results in a considerable proportion of PC being identified only retrospectively when recurrences ensue ​[8]. 

In this patient, multiple features predicted a probability of malignant disease - symptomatic hypercalcemia with bone and renal involvement, anemia, peptic disease, very high levels of PTH and ALP, voluminous parathyroid gland, and apparent invasion of adjacent structures on CT. The treatment of choice in parathyroid crisis is urgent parathyroidectomy, but such could not be performed promptly, since a specialized team was not available at our hospital. Renal dysfunction represented an additional challenge in the acute management of hypercalcemia since zoledronate is contraindicated and pamidronate could cause a further decline in kidney function. The choice was to use cinacalcet, which proved to be effective in lowering calcemia and safe in the setting of renal dysfunction. 

However, intraoperative findings were discrepant so, in the absence of aspects suggestive of malignancy, including on frozen section analysis, the choice was made to perform simple parathyroidectomy, successfully avoiding the unnecessary risk of a more extensive surgery.

Despite the benign nature of the lesion, this GPA still presented in a severe, life-threatening way with parathyroid crisis and was responsible for significant morbidity. Bone findings and sustained clinical cues suggested a prolonged disease and a blood work compatible with PHPT was obtained months prior. Had the diagnosis been promptly established and treatment ensured, the medical emergency of a parathyroid crisis could have been averted, as well as additional morbidity from the latest fracture. 

## Conclusions

Definitive diagnosis of PC is challenging, as many clinical, imagiological, and laboratory features overlap with benign PA, especially with GPA. Parathyroid crisis is a rare but potentially life-threatening complication of hypercalcemic PHPT presenting with multiorgan failure, and clinical suspicion is essential for timely diagnosis and treatment. Ionized calcium evaluation might be useful whenever serum calcium levels are discordant with clinical features of hypercalcemia.
